# Role of CD147 in the development and diagnosis of hepatocellular carcinoma

**DOI:** 10.3389/fimmu.2023.1149931

**Published:** 2023-04-06

**Authors:** Defa Huang, Dingyu Rao, Qing Jin, Mi Lai, Jiali Zhang, Zhonghong Lai, Haibin Shen, Tianyu Zhong

**Affiliations:** ^1^ Laboratory Medicine, First Affiliated Hospital of Gannan Medical University, Ganzhou, China; ^2^ Department of Cardiothoracic Surgery, First Affiliated Hospital of Gannan Medical University, Ganzhou, China; ^3^ The First School of Clinical Medicine, Gannan Medical University, Ganzhou, China; ^4^ Department of traumatology, First Affiliated Hospital of Gannan Medical University, Ganzhou, China; ^5^ Precision Medicine Center, First Affiliated Hospital of Gannan Medical University, Ganzhou, China

**Keywords:** hepatocellular carcinoma, CD147, metastasis, angiogenesis, diagnosis

## Abstract

Hepatocellular carcinoma (HCC) is the most common primary liver cancer, and the third leading cause of cancer-related deaths worldwide. HCC is characterized by insidious onset, and most patients are diagnosed at an advanced stage with a poor prognosis. Identification of biomarkers for HCC onset and progression is imperative to development of effective diagnostic and therapeutic strategies. CD147 is a glycoprotein that is involved in tumor cell invasion, metastasis and angiogenesis through multiple mechanisms. In this review, we describe the molecular structure of CD147 and its role in regulating HCC invasion, metastasis and angiogenesis. We highlight its potential as a diagnostic and therapeutic target for HCC.

## Introduction

Liver cancer, one of the most common malignant tumors in humans, is mainly divided into primary and secondary subgroups. Primary liver cancer is ranked seventh and second with regards to incidence and mortalities, respectively worldwide (1). Hepatocellular carcinoma (HCC), the leading type of primary liver cancer, is the third leading cause of cancer-related deaths worldwide, killing 745,500 patients each year (2, 3). In Asia, chronic hepatitis B virus (HBV) infection is the leading cause of HCC (4), whereas chronic hepatitis C virus (HCV), alcoholic cirrhosis and steatohepatitis are the main causes across Western countries (5). Other risk factors for HCC include heavy alcohol consumption, aflatoxin ingestion, obesity, type 2 diabetes and smoking (6, 7). Despite advances in diagnosis and treatment strategies, prognosis of HCC patients remains unsatisfactory, with a 5-year survival rate of only 15-20%, a rate that has changed little in the past 30 years (8, 9). These minute changes have been attributed to lack of reliable early biomarkers and the high economic challenge of effective treatment in countries with high risk factors (10). Current treatment modalities for HCC mainly involve surgical interventions, such as ablation, resection and organ transplantation (11, 12). However, these treatment approaches are often limited by late diagnosis, coupled with lack of transplantable organs or disease that progress beyond the Milan criteria (13). Therefore, urgent identification of novel molecular mechanisms and diagnostic markers is imperative to development of strategies for effective treatment of HCC.

Cluster of differentiation 147 (CD147), a glycoprotein originally known as a regulator of Matrix metalloproteinase (MMP), serves as a potential target for cancer therapy through cell-matrix and cell-cell interactions (14, 15). Studies have shown that CD147 is not only overexpressed in cancer cells, but also regulates cell proliferation, drug resistance and cell stromal adhesion properties (16–18). Earlier reports indicated that apart from regulating MMP, CD147 also plays a role in several other functions, and can also bind different molecular partners to regulate multiple signaling pathways (19, 20). In addition, CD147 is involved in angiogenesis by regulating production of vascular endothelial growth factor (VEGF) in tumor and stromal cells (21). In addition, CD147 acts on cancer-associated fibroblasts to promote tumorigenesis and development. It was found that CD147 is expressed on melanoma cells and induces tumor cell invasion by stimulating fibroblast production of matrix metalloproteinases (22). Xu et al. (23)found that CD147 transformed breast cancer static fibroblasts into cancer-associated fibroblasts.

Prospecting for novel mechanisms regulating HCC development, coupled with early HCC detection, can greatly improve chances of effective treatment. Studies have described the role of new diagnostic biomarkers in clinical and therapeutic management of HCC (24). Notably, numerous evidence indicates that CD147 is a promising diagnostic and therapeutic biomarker for HCC (25).

## CD147 structure

**Figure 1:**
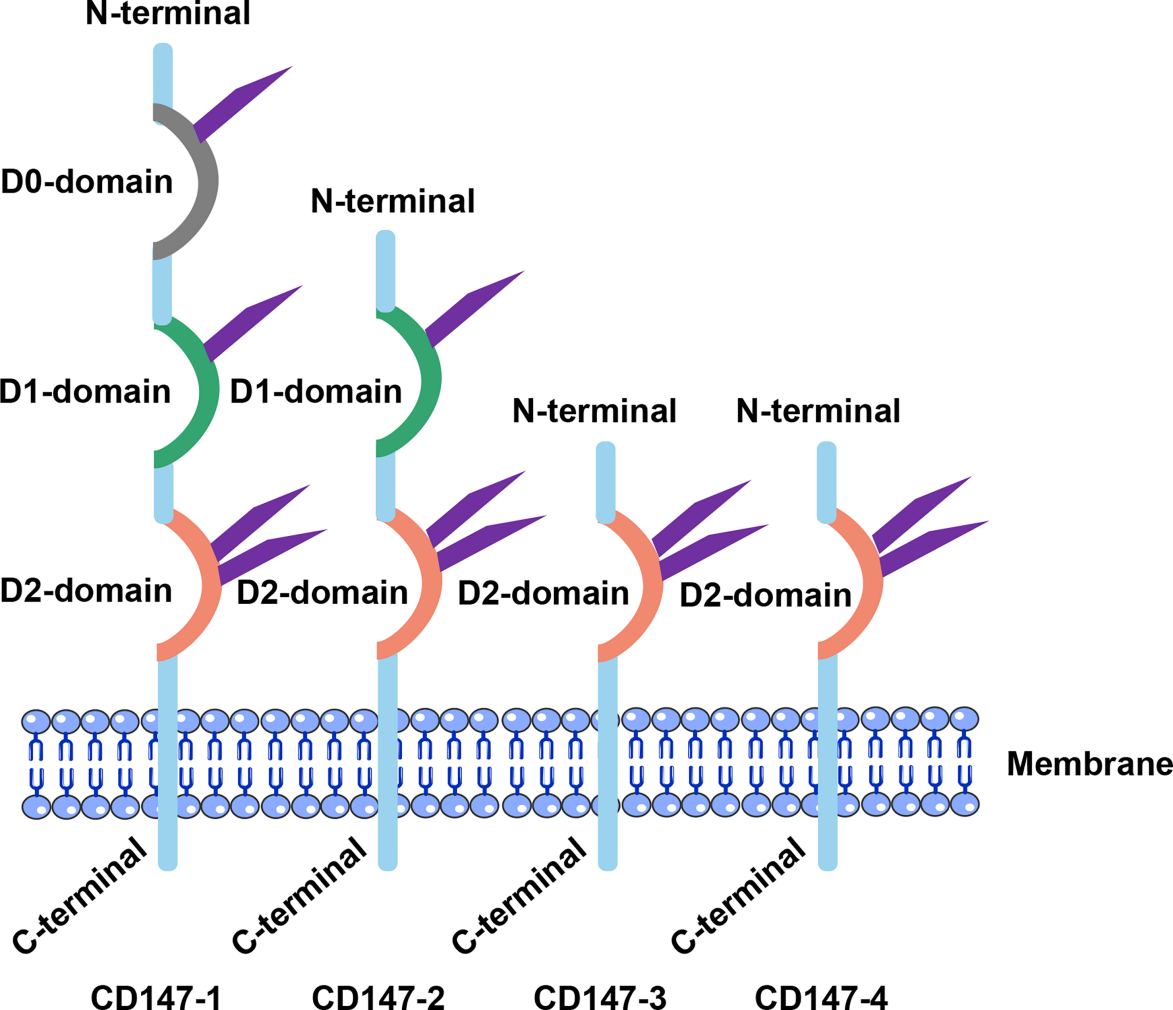
Structural characteristics of CD147. CD147’s extracellular segments differed significantly. D0: retina-specific structural domain; D1: Ig C2 structural domain; D2: Ig I structural domain.

CD147 which plays numerous functions, was given different names by different researchers during early days, including gp42, BSG, and EMMPRIN, among others (26–28). The Human Genome Project uses the name BSG, while its corresponding gene and protein name is basigin (Ok blood group) (20). Apart from detection in all vertebrates. This gene is also homologous in *Drosophila melanogaster* and Schistosoma (29). The gene encoding CD147 is located on chromosome 19, p13.3, and comprises 10 exons on a ~12 kb fragment (30). A 30 bp element, located at the -142 to -112bp 5’ end of the gene, contains binding sites for specificity protein 1 (Sp1), activator protein 1 (AP1), transcription factor II (TFII) and early growth response factor-2, which are important for CD147 transcription (31, 32). The 3’ flanking region also has two hypoxia-inducible factor (HIF) binding sites (33, 34). Human Protein Database shows that four variants of CD147 has been encoded through an alternative promoter and splicing (35, 36) (**Figure 1**). The Ig-like structural domain is divided into four types, namely type V, C1, C2, and I. Notably, the latter type lies between types V and C. Moreover, CD147-1 has three Ig-like structural domains and is a retina-specific type (37, 38). CD147-2, a common classical isoform that is widely distributed, has two Ig-like structural domains and three asparagine-glycosylated aspartate sequence sites (39, 40). Structurally, one monomer of CD147 is composed of two domains, D1 corresponding to N-terminal domain and a C-terminal domain called D2 (**Figure 1**). On the other hand, CD147-3 and CD147-4 are less common and contain only 1 extracellular structural domain (Ig I). Studies have shown that CD147-3 can act as an endogenous inhibitor of CD147-2 by forming a heterodimer with it (35). Notably, the transmembrane region of BSG proteins comprises 23 amino acids that are highly conserved in the CD147 family as well as across species (41, 42). A fully conserved Glu in the middle of the transmembrane region is of particular interest, owing to the fact that it may not only mediate CD147 interactions with other adjacent proteins in the membrane but also regulate important CD147 functions. Moreover, the transmembrane region contains a typical leucine zipper structural domain that is involved in both membrane-protein interactions and multiple intracellular signaling pathways (41, 43).

Studies have shown that CD147 interacts with numerous partners, such as caveolin-1 (44), monocarboxylate transporter (45), CD98 and β1 integrin (46), to promote various processed including cell metabolism, proliferation, migration and invasion (47). In addition, the soluble form of CD147 was found to internalize and promote cell proliferation and migration through surface CD147 binding (48, 49). Another study demonstrated that CD147 overexpression mimics VEGF production through the PI3K/AKT signaling pathway, thereby directly promoting tumor angiogenesis (50). Moreover, Chen et al. (51) reported that CD147 was overexpressed in human umbilical vein endothelial cells, while its upregulation by specific siRNAs markedly suppressed angiogenesis in multiple ways, including proliferation, migration, secretion of MMPs, and activation of the PI3K/Akt pathway. It has been shown that CD147 is associated with the development of various solid tumors such as esophageal cancer, head and neck squamous cell carcinoma, oral squamous cell carcinoma, gastric cancer, colorectal cancer, and breast cancer (52–72). The biological roles of CD147 in different cancers are shown in **Table 1**.

**Table 1:** The biological roles of CD147 in different cancers.

Cancer type	Biological role	References
Esophageal Squamous Cell Carcinoma	Inhibits proliferation, invasion, and angiogenesis; prognostic predictors; potentially therapeutic target	(52–55)
Esophageal Adenocarcinoma	Potential biomarkers	(56)
Head and Neck Squamous Cell Carcinoma	Promote proliferation and metastasis; potential prognostic and treatment biomarker	(57, 58)
Oral Squamous Cells Carcinoma	Promote proliferation, invasion, angiogenesis;	(59–61)
Pancreatic	Induced pancreatic cancer cell invasiveness; mediated cellular resistance	(62–64)
Gastric cancer	Indicator of tumor recurrence and prognosis; promote invasion, angiogenesis	(60, 65–67)
Colorectal cancer	Promote Invasion; Biomarker	(68–70)
Breast cancer	Inhibits proliferation, invasion; mediates chemoresistance; predicted prognosis	(71, 72)

## CD147 promotes HCC invasion and metastasis

**Figure 2:**
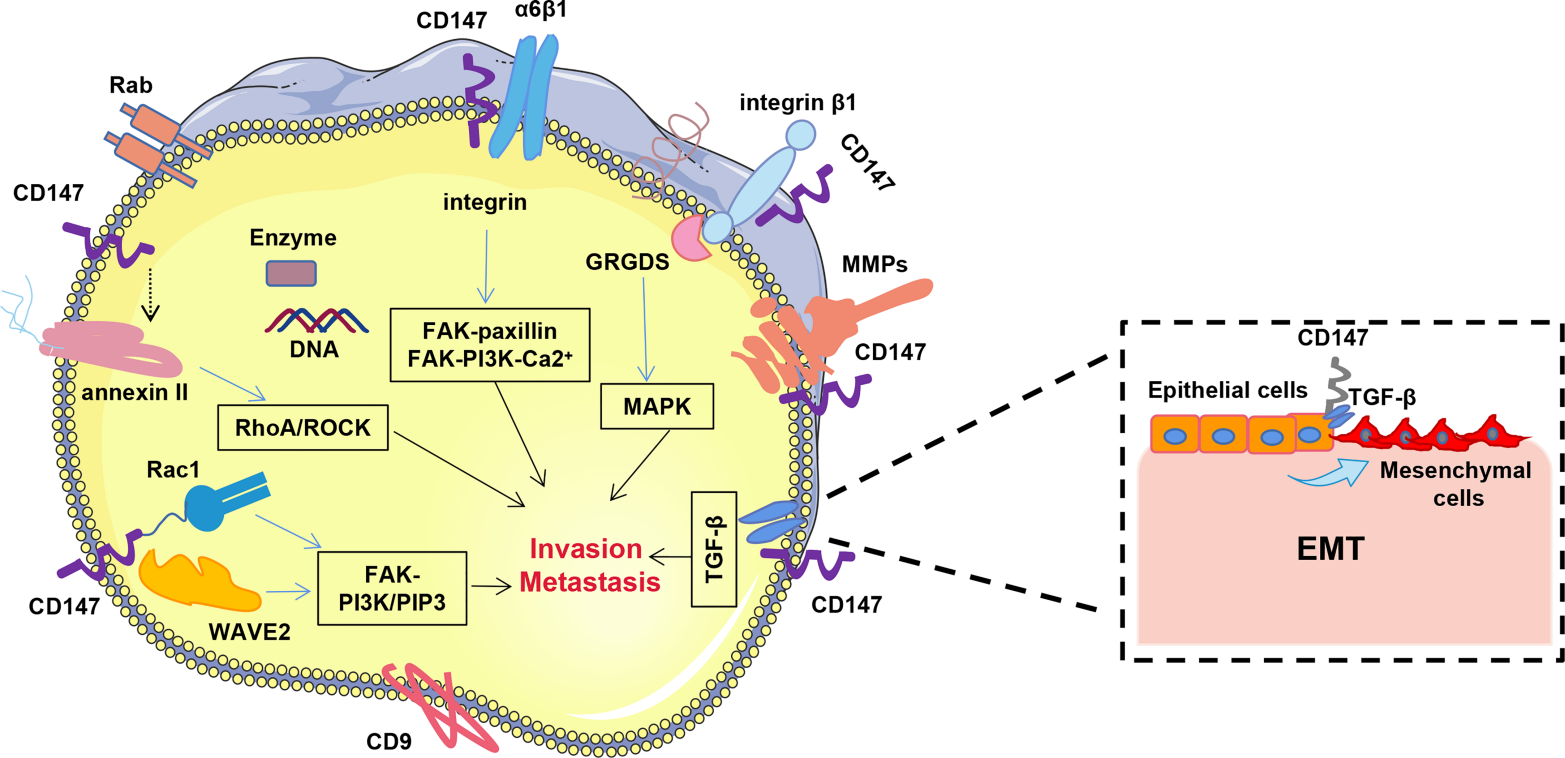
CD147 promotes hepatocellular carcinoma invasion and metastasis. CD147 promotes HCC invasion and metastasis mainly through FAK-PI3K-Ca2^+^, RhoA/ROCK, FAK-PI3K/PIP3,TGF-β and MAPK signaling pathways.

Tumor invasion relies on a complex mechanism that includes cell adhesion, migration, and stromal degradation. CD147 enrichment on the surface of tumor cells is an important regulator of tumor mesenchymal interactions, as it stimulates the neighboring mesenchyme to promote synthesis of several MMPs (mainly MMP-2,9). These enzymes degrade the extracellular matrix composed of collagen, elastin, adhesion proteins and proteoglycans, thereby providing conducive conditions for cell metastatic movement (73–75). Previous studies have shown that CD147’s extracellular N-terminal region is critical for MMP induction (73, 76). The epithelial-mesenchymal transition (EMT) is a key developmental program that is often activated during cancer

invasion and metastasis. Ru et al. (77) demonstrated that CD147 plays an important role in invasion by promoting EMT of hepatocytes through the TGF-β signaling pathway (**Figure 2**).

Cell motility plays a crucial role in tumor invasion and metastasis. Notably, CD147 promotes HCC invasion and metastasis through several different pathways, including integrin-mediated FAK-paxillin, FAK-PI3K-Ca2^+^, RhoA/ROCK, WAVE2, Rac1 and MAPK signaling pathways (**Figure 2**). Studies have shown that CD147 co-localizes with integrin α3β1 and α6β1 in hepatocellular carcinoma cells and mediates FAK-paxillin as well as FAK-PI3K-Ca2^+^ signaling pathways through their interaction to promote both invasive and metastatic potential of HCC cells (75, 78). Binding of CD147 to the integrin β1 subunit competitively prevents its binding to the GRGDS peptide, leading to cytoskeletal rearrangement (47). Conversely, CD147 inhibits the RhoA/ROCK signaling pathway and amoeboid motility in HCC cells by attenuating annexin II phosphorylation. Moreover, it also promotes localization of the Verprolin homolog 2 (WAVE2) membrane and activation of Rac1 in HCC cells *via* the integrin-FAK-PI3K/PIP3 signaling pathway, thereby contributing to formation of amoeboid and mesenchymal motility (79). Cui et al. (80) demonstrated that CD147 dimerization is essential for the induction of hepatocellular carcinoma MMPs and cell invasion *via* the MAPK pathway. Additional evidence has shown that membrane-linked protein II promotes HCC invasion and metastasis *in vitro* by interacting with CD147 (81).

## CD147 promotes HCC angiogenesis

**Figure 3:**
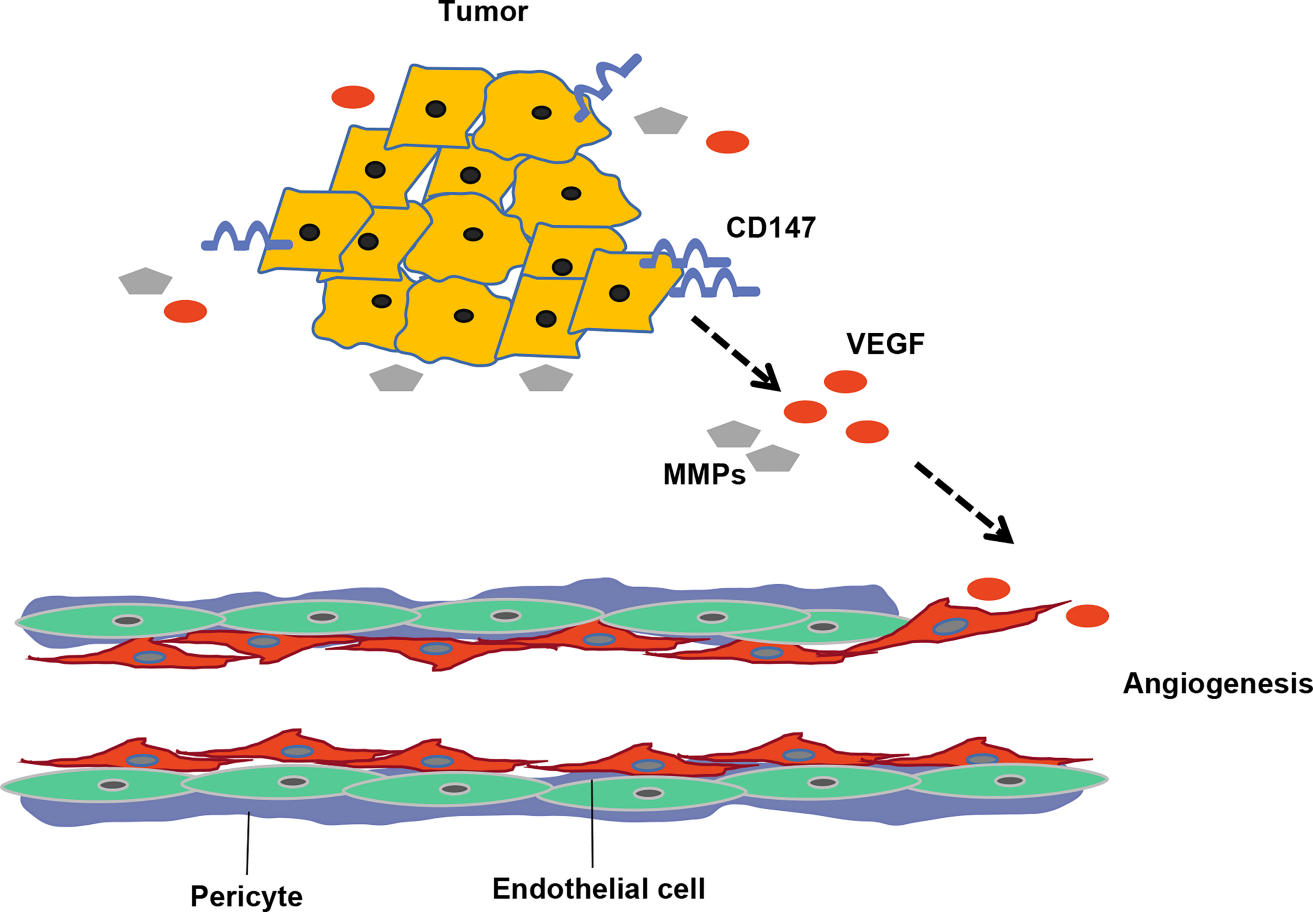
CD147 promotes hepatocellular carcinoma angiogenesis. CD147 enhances production of MMPs and VEGF thereby promoting HCC angiogenesis.

Studies have shown that CD147 is also involved in tumor angiogenesis, a key component of the tumor microenvironment. CD147-induced MMP expression in tumor and stromal compartments subsequently mediates release of biologically active angiogenic growth factors from stromal binding complexes (82)(**Figure 3**). Tang et al. (83) demonstrated that CD147 stimulated tumor angiogenesis by upregulating VEGF and MMP expression in tumor and mesenchymal compartments. Results from both *in vitro* and *in vivo* tumor models indicated that tumor CD147 promoted production of endothelial VEGF soluble isoforms (especially the most angiogenic ones) and their major receptor VEGFR-2 *via* transcription factor HIF-2α (84). In addition, CD147 promotes capillary-like formation *via* VEGFR-2 and its ligand VEGF (21). A disintegrin and metallo-proteinases (ADAM) family of protein hydrolases is anchored to the cell membrane, is broadly expressed, evolutionarily conserved, and is the main enzyme involved in the extramembrane cleavage of molecules (85). It has been reported that ADAM12 cleaved the extracellular segment of CD147 and fully bound the free CD147 to the receptor cells, thus regulating tumor angiogenesis (86). Wu et al. (87)found that ADAM10 decomposes CD147 to produce cytoplasmic fragments and promotes HCC development by promoting autophagy.

Remodeling of the tumor microenvironmental matrix by VEGF and MMPs is essential for angiogenesis. Studies have shown that CD147 expression is positively correlated with VEGF, MMP-2, MMP-9 and microvessel density CD34 (MVD-CD34) expression in HCC tissues (88, 89). Results from *in vitro* and *in vivo* experiments showed that interfering with CD147 expression in mouse hepatocellular carcinoma cells not only significantly downregulated MMP-11 and VEGF-A expression at both mRNA and protein levels, but also suppressed invasiveness, adhesion and metastasis to lymph nodes (90). Another study demonstrated that Kaposi’s sarcoma-associated herpes virus (KSHV) promotes invasiveness of fibroblasts and endothelial cells by upregulating CD147 (91), while enhanced invasiveness of KSHV-infected endothelial cells was attributed to activation of VEGF through CD147-dependent PI3K/AKT and MAPK (92). Collectively, these data indicate that CD147 promotes angiogenesis by directly regulating secretion of MMP and VEGF on the one hand, and inducing HCC invasion by activating VEGF through the CD147-dependent PI3K/AKT and MAPK signaling pathways on the other.

## CD147 as a diagnostic biomarker for HCC

Numerous studies have highlighted the significance of CD147 in tumor progression, thus affirming its role in tumor diagnosis. CD147 is overexpressed in a variety of cancers, such as lung, breast, prostate, stomach, and genitourinary cancers (93–97). Another study suggested that CD147 may be an important independent predictor of poor survival in HCC patients, owing to its role in tumor growth, invasion and angiogenesis (98). The results showed that the expression of CD147 was positively correlated with metalloproteinase-2, vascular endothelial growth factor and microvascular density CD34 in hepatocellular carcinoma patients. Patients with high CD147 expression had poor survival (98). Given the important role played by CD147 in tumor cell growth, survival and invasive metastasis, coupled with its widespread expression in human malignancies, researchers have employed proteomics techniques to analyze differential expression of proteins in liver cancer plasma/serum. Results indicate that CD147 antigen is specifically highly expressed in the plasma of liver cancer patients. Zhu et al. (99) suggested that the higher the expression of CD147 or the better the degree of tumor differentiation, the longer the survival of patients with liver cancer, thus an effective therapeutic target for interfering with or reversing HCC progression. On the other hand, Wu et al. (100) found that serum soluble CD147 levels were not only significantly higher in HCC patients than healthy subjects, but were also associated with tumor size and Child-Pugh classification. that the authors concluded that detection of soluble CD 147 has some value in HCC diagnosis. In addition, CD147 expression is strongly correlated with HCC prognosis. oliver et al. (101) demonstrated that HCC patients with low CD147 expression had longer survival. However, a meta-analysis by Peng et al. showed no correlation between HCC survival and CD147 expression (102).Taken together, these studies indicate that CD147 plays a crucial role in HCC progression, and affirm its potential as a diagnostic biomarker.

## CD147 as a therapeutic target for HCC

The role of CD147 in tumorigenesis has made it a new target for development of tumor therapies. The basic approach for targeted therapy entails down-regulating expression of CD147 protein *via* RNAi technology (103), small molecule compounds (104), anti-CD147 monoclonal antibodies (105, 106) or polyclonal antibodies (107) with the aim of blocking CD147 function. Multiple antigenic peptide vaccines have also been employed (108). To target CD147, the therapeutic agent Licartin (generic name (I131) metuximab injection) was developed as an anti-CD147 monoclonal antibody HAb18 coupled to the radioisotope I131. Results of a Phase I/II trials demonstrated that Licartin is safe, thus it was officially approved for clinical use by the Chinese State Food and Drug Administration (SFDA, registration number S20050039) (109). Results from a randomized trial showed that Licartin prevents HCC recurrence after liver transplantation (110). Despite Licartin’s efficacy in HCC, its clinical application has been limited by radioactive I131 component. Wang et al. (111) experimentally tested four anti-CD147 antibodies in HCC, and found that while 1B 3 and 3B 3 effectively inhibited MMP-2 secretion and cell invasion, HAb 18 Gedomab 1 and HAb18 Gedomab 2 exhibited opposite effects. Wang et al. developed an anti-CD147 antibody- HAb 18. The chimeric antibody cHAb 18 contains variable heavy and light chains of HAb 18 antibody and a constant region of human IgG 1 γ1. The authors found that cHAb 18 treatment not only effectively suppressed liver tumor metastasis but also prolonged survival in an *in situ* HCC mouse model (112). Apart from the anti-CD 147 antibody strategy, researchers have demonstrated that small molecule (AC-73) inhibitors of CD147 dimerization can suppress MMP-2 production in hepatocellular carcinoma *via* the CD147-ERK-STAT 3-MMP-2 signaling pathway (104). Tseng et al (113). used chimeric antigen receptor therapy with CAR-transduced T and NK cells that recognize the surface marker CD147 to effectively kill various malignant HCC cell lines *in vitro*, as well as HCC tumors in xenograft and patient-derived xenograft mouse models. These findings support the therapeutic potential of CD147-CAR-modified immune cells for HCC patients.

## Discussion

CD147 is a cell adhesion molecule involved in intercellular and extracellular matrix interactions. Functionally, it stimulates secretion of MMP without affecting production of tissue inhibitors of metalloproteinases (physiological inhibitors of MMP), thereby altering the collagenolytic balance to activate MMP (114). Members of the CD147 family widely differ in molecular weights, depending on the species, tissues and cells (115–117). Studies have shown that CD147 is not only highly expressed in HCC, but is also closely associated with its development (100). Notably, CD147 promotes HCC invasion and metastasis through integrin-mediated FAK-paxillin, FAK-PI3K-Ca2^+^, RhoA/ROCK, WAVE2 and Rac1 signaling pathways. In addition, CD147 can induce VEGF and MMPs formation to promote HCC angiogenesis. In this review, we have described the CD147 structure and its underlying molecular mechanism in HCC invasion, metastasis and angiogenesis. In addition, we have highlighted its potential as a diagnostic marker for HCC. HCC develops due to accumulation of multiple factors and interaction of multiple mechanisms. In addition, CD147 plays a role in the immune infiltration or immune escape of the tumor microenvironment. Chen et al (118). showed that CD147 regulates anti-tumor CD8 T cell responses to promote tumor immune escape. In recent years, extracellular vesicles (EVs) have been extensively studied. Study finds CD14-positive EVs are a novel biomarker of HCC and cholangiocarcinoma liquid biopsy that permit a non-invasive assessment of the presence and possible extent of these cancers in patients with advanced liver diseases (119). Yang et al (120). noted that immune cells- derived EVs containing integrin αMβ2 or CD147 may facilitate HCC metastasis.

Currently, there is no effective therapy available to treat HCC. Sorafenib is a widely used first-line standard agent for the treatment of advanced HCC, but has been shown to have low efficacy and severe side effects (121). Opdivo, a PD-1 blocker, has been approved by the U.S. Food and Drug Administration as a second-line treatment strategy for HCC patients previously treated with Sorafenib (122). CD147 is being investigated as a new target for HCC treatment. Anti-CD147 monoclonal antibody-targeted therapy for HCC is a promising strategy. Cost of use and security is a major challenge. Overcoming these problems will make CD147 prominent in the treatment of HCC. Notably, prognosis of HCC patients has seen little improvement in the last two decades, possibly due to limited information on the molecular mechanisms underlying its progression. Urgent elucidation of these mechanisms is imperative to future development of novel and effective therapeutic strategies and reliable diagnostic biomarkers for HCC patients.

## Author contributions

DH, DR, and QJ searched for literature and wrote the first draft of this article. ML and JZ edited the manuscript. ZL edited figures. HS and TZ strictly reviewed the manuscript and polished the grammar. All authors contributed to the article and approved the submitted version.
